# Optimizing performance of nonparametric species richness estimators under constrained sampling

**DOI:** 10.1002/ece3.2463

**Published:** 2016-09-22

**Authors:** Harshana Rajakaruna, D. Andrew R. Drake, Farrah T. Chan, Sarah A. Bailey

**Affiliations:** ^1^ Great Lakes Laboratory for Fisheries and Aquatic Sciences Fisheries and Oceans Canada Burlington ON Canada; ^2^ Department of Biological Sciences University of Toronto Scarborough Toronto ON Canada

**Keywords:** abundance‐based estimator, biodiversity statistics, Chao, community ecology, incidence‐based estimator, Jackknife, rare species

## Abstract

Understanding the functional relationship between the sample size and the performance of species richness estimators is necessary to optimize limited sampling resources against estimation error. Nonparametric estimators such as Chao and Jackknife demonstrate strong performances, but consensus is lacking as to which estimator performs better under constrained sampling. We explore a method to improve the estimators under such scenario. The method we propose involves randomly splitting species‐abundance data from a single sample into two equally sized samples, and using an appropriate incidence‐based estimator to estimate richness. To test this method, we assume a lognormal species‐abundance distribution (SAD) with varying coefficients of variation (CV), generate samples using MCMC simulations, and use the expected mean‐squared error as the performance criterion of the estimators. We test this method for Chao, Jackknife, ICE, and ACE estimators. Between abundance‐based estimators with the single sample, and incidence‐based estimators with the split‐in‐two samples, Chao2 performed the best when CV < 0.65, and incidence‐based Jackknife performed the best when CV > 0.65, given that the ratio of sample size to observed species richness is greater than a critical value given by a power function of CV with respect to abundance of the sampled population. The proposed method increases the performance of the estimators substantially and is more effective when more rare species are in an assemblage. We also show that the splitting method works qualitatively similarly well when the SADs are log series, geometric series, and negative binomial. We demonstrate an application of the proposed method by estimating richness of zooplankton communities in samples of ballast water. The proposed splitting method is an alternative to sampling a large number of individuals to increase the accuracy of richness estimations; therefore, it is appropriate for a wide range of resource‐limited sampling scenarios in ecology.

## 1 Introduction

Species richness, also known as alpha diversity, is the simplest way of characterizing the diversity of ecological communities (Gotelli & Colwell 2011). Quantifying richness informs numerous conservation objectives, such as measuring extinction rates (Colwell & Coddington, 1995) and projecting the number of species likely to invade a new habitat (e.g., Lockwood et al. 2009). The ability to count every organism in a community would allow for exact measures of species richness; yet, this is rarely possible even in finite communities due to logistical counting constraints. Therefore, richness estimation is chiefly a statistical problem, which involves estimating the classes in a statistical population based on one or more samples (Chao, 1984; Colwell & Coddington, 1995).

The number of observed species in a sample regularly underestimates local richness due to the sampling effect of failing to document rare species in the community that exist at levels below a “singleton” in the sample (Colwell & Coddington, 1995). This characteristic leads to sample‐based measures of richness that are intrinsically negatively biased (Chao, 1984; Brose, Martinez, & Williams, 2003; Walther & Moore 2005). Like most statistical estimates, the accuracy of a richness estimate increases with sampling intensity by increasing sample coverage (Brose et al., 2003; Walther & Moore 2005); however, because sampling resources to physically enumerate communities are almost always limited (financial cost of surveys, time required for sample collection, processing, and identification), methods to optimize richness estimation against sampling effort have been the subject of considerable investigation (e.g., Basualdo, 2011; Brose et al., 2003; Colwell & Coddington, 1995; Walther & Morand, 1998).

Numerous species richness estimators have been developed to provide repeatable and standardized estimates of the true underlying richness of communities based on sample data (Brose et al., 2003). Estimators have the desirable property of accounting for the undetected (i.e., rare) species in an assemblage; common estimators include homogeneous models, parametric and Bayes models, and nonparametric methods (see reviews in Bunge & Fitzpatrick, 1993; and Colwell & Coddington, 1995). While there is no clear consensus as to which estimator performs universally best (e.g., Basualdo, 2011; Brose et al., 2003; Walther & Morand, 1998), some authors suggest that nonparametric methods perform better than iterative estimators such as species accumulation curves, or parametric methods in general (Brose et al., 2003). Nonparametric methods project the total number of species in the statistical population based on the total species observed in the sample, plus a correction involving the number of rare species (typically, based on singletons or doubletons; Colwell & Coddington, 1995) in the sample to account for the unobserved fraction of rare species in the statistical population. Nonparametric approaches are popular because they do not require assumptions about the relative abundance of species in communities (Chao 2005) and perform relatively well under realistic scenarios, especially for communities with a large membership of singletons and doubletons (Colwell & Coddington, 1995; Walther & Moore 2005).

Nonparametric estimators can be divided into two general classes. The first class, termed abundance‐based estimators, can be applied to counts of individuals for each species in a single sample (i.e., abundance data) and commonly includes the Chao1 (Chao, 1984), abundance‐based Jackknife (1st, 2nd…kth order) (Chiu, Wang, Walther, & Chao, 2014), and abundance‐based coverage estimator (ACE) (Gotelli & Colwell, 2011). The second class, termed incidence‐based estimators, can be applied to presence or absence (i.e., incidence) data based on a set of replicate samples. By default, incidence‐based estimators rely on a series of species discovery matrices and include the Chao2, incidence‐based Jackknife (1st, 2nd…kth order) (Chiu et al., 2014) and incidence‐based coverage estimator (ICE) (Gotelli & Colwell, 2011). Because abundance‐ and incidence‐based approaches use different sampling schemes (abundance‐based sampling, where the abundance of all species in a single sample is recorded vs. incidence‐based sampling, where the incidence of all species across at least two samples is recorded), understanding the relative performance of each class of estimator for a given allocation of sampling resources can be used to optimize the choice of the estimator, thereby reducing the estimation error in resource‐limited scenarios. In particular, quantifying the performance of nonparametric estimators under abundance‐ vs. incidence‐based sampling can provide practical guidance for large‐scale species monitoring programs where the choice of abundance vs. incidence sampling can substantially influence sampling time over potentially hundreds of sampling events.

Our aim was to evaluate the performance of nonparametric estimators and provide an unbiased comparison of abundance‐ vs. incidence‐based approaches by randomly splitting a single species‐abundance sample and conducting a richness estimation using an incidence‐based estimator vs. estimation with the single species‐abundance sample using an abundance‐based estimator to increase the estimation accuracy. By doing so, we contribute to the existing literature on the performance of species richness estimators with an emphasis on resource‐limited sampling scenarios involving small sample sizes compared to population sizes. We focus primarily on lognormal species‐abundance distributions (SADs), which are considered common in many ecological communities (Preston, 1948; Hubbell, 2001; McGill, 2003; Connolly, Hughes, Bellwood, & Karlson, 2005; Dornelas & Connolly 2008), and secondarily on log series, geometric series, and negative binomial species‐abundance distributions (see McGill et al., 2007). In such way, we observe the effect of the estimator choice and sampling scheme across a range of community types and the resulting levels of estimation accuracies. Thus, we evaluate the performance of each estimator in relation to the predominance of rare species, thereby providing structured guidance, on the basis of mathematical and statistical criteria, as to how to decide between the estimators to obtain the most accurate richness estimations. We illustrate the application of the proposed methodology, and advantages therein, for estimating species richness of zooplankton communities in samples of ballast water.

## 2 Methods

### 2.1 Performance criteria: mean‐squared error (MSE)

We evaluated nonparametric richness estimators (abundance‐based: Chao1 (bias‐corrected form), Jackknife1a and 2a (Jk1a/2a) (i.e., 1st and 2nd order), and ACE; incidence‐based: Chao2 (bias‐corrected form), Jackknife1i/2i (Jk1i/2i) (i.e., 1st or 2nd order—both are theoretically the same for two sample splits), and ICE (from Gotelli & Colwell 2004; Chiu et al., 2014) using Markov chain Monte Carlo (MCMC) samples simulated from lognormal, log series, geometric series, and negative binomial SADs in the following approach. Note that we used the bias‐corrected version of Chao1 and Chao2 estimators in order to accommodate both the presence and absence of singleton (i.e., species represented by exactly one individual) and doubletons (i.e., species represented by exactly two individuals) (Chao 2005; Chiu et al., 2014).

Suppose that we obtain *k* repeated, independent, identically distributed samples, $\left(X^{\left(1\right)},\ldots ,\;X^{\left(k\right)}\right)$, with replacement (“with replacement” recommended by Walther & Moore 2005) from a statistical population, which consists of species *s*_*i*_ for *i *=* *1,2,…,*S*, such that *S* is defined as the species richness parameter, and the abundance corresponding to each species is given by *b*_*i*_, such that the total abundance of the statistical population is given by $N=\sum_{i=1}^{S}b_{i}$. Thus, the expected value of a richness estimator, $\theta_{S}\in R$, that is, ${\hat{\theta }}_{S}\left(X^{\left(1\right)},\ldots ,X^{\left(k\right)}\right)$ for a number of repeated samples *k,* should tend toward true population richness *S* for the estimator to be unbiased. That is, we define bias as $\text{bias}\left({\hat{\theta }}_{S}\right)=E\left({\hat{\theta }}_{S}\right)-S$. The expected mean‐squared error (MSE), here described generically as “accuracy,” is given by $\text{Var}\left({\hat{\theta }}_{S}\right)+{\text{bias}}^{2}\left({\hat{\theta }}_{S}\right)$, such that it accounts also for the variance (precision) of the estimates of $\theta_{S}$, $\text{Var}({\hat{\theta }}_{S})$, around the expected value ${\hat{\theta }}_{S}$, which is given by $E({\left|\right|{\hat{\theta }}_{S}-S\left|\right|}^{2})$ (see also Walther & Moore 2005). Therefore, MSE indicates the likelihood that a random sample will deviate from the true population parameter value *S*.

### 2.2 Derivation of the probability model for MCMC sampling

Here, we assume SADs are lognormal. Appendix S1 shows that lognormal distributions are the most common type of zooplankton SAD across 156 independent samples of ballast water. The knowledge that SADs are lognormal cannot be used directly to generate theoretical samples using Markov chain Monte Carlo (MCMC) simulations without converting the distribution to a frequency distribution of abundance by species, which can then be converted to a probability distribution model of catchability (i.e., a probability of each species being caught in a sample) for sampling. Therefore, following the cumulative density function of a lognormal SAD, we can write the number of species having log‐abundance from ‐∞ to ln *N* as,

(1) $$s_{\ln\;N}=\frac{S}{\sigma \sqrt{2\pi }}\int_{-\infty }^{\ln\;N}\text{exp}\left[-\frac{1}{2}{\left(\frac{\text{ln}X-\mu }{\sigma }\right)}^{2}\right]\text{dln}X=\frac{S}{2}\left[1+\text{erf}\left(\frac{\text{ln}N-\mu }{\sqrt{2}\sigma }\right)\right]$$

Here, *S* is the number of species in the assemblage; *N* is the total number (i.e., abundance) of individuals of all species in the assemblage (statistical population); μ is the mean and σ is the standard deviation, given by μ = ln(mean) − var/2, and $\sigma^{2}=\text{ln}\left|\left(1+{\text{var/mean}}^{2}\right)\right|$, where mean and var are the mean abundance per species and the variance given by (CV·mean)^2^, respectively, of the non‐log‐transformed statistical populations (where CV is the coefficient of variation); and erf is the error function (Abramowitz & Stegun, 1972). Note that *S* is given by $\int_{-\infty }^{+\infty }\frac{S}{\sigma \sqrt{2\pi }}\text{exp}\left[-\frac{1}{2}{\left(\frac{\text{ln}N-\mu }{\sigma }\right)}^{2}\right].$

Thus, we note that 1 deducted by the inverse of Equation (1) yields the log‐abundance by ranked‐species (*s*_*i*_) from the most common to the rarest, in the order of *s*_*i*_ = 1…*S*, as,

(2) $$\text{ln}N_{s_{i}}=1-\sigma \sqrt{2}{\text{erf}}^{-1}\left(\frac{2s_{i}}{S}-1\right)-\mu$$

Note that the quantity $\frac{s_{i}}{S}$ varies from >0 to 1 and each species *s*_*i*_ is represented by the fraction $\frac{s_{i}}{S}$ in order of their abundance from the most common to the rarest; *s*_*i*_ = 1…*S*. Assuming that the probability of an individual being selected (sampled) from the statistical population of a species assemblage is independent of species identity (i.e., selection is equally likely across species), we can transform Equation (2) into a probability distribution by taking the exponential of the equation and normalizing it by total abundance. This leads to the probability of an individual being selected (sampled) from the species assemblage and belonging to ranked‐species s_i_ as,

(3) $$\mathrm{Pr}\left(s_{i}\right)=\frac{\text{exp}\left(-\sigma \sqrt{2}\left[{\text{erf}}^{-1}\left(\frac{2s_{i}}{S}-1\right)\right]\right)}{\sum_{0}^{1}\text{exp}\left(-\sigma \sqrt{2}\left[{\text{erf}}^{-1}\left(\frac{2s_{i}}{S}-1\right)\right]\right)},$$

for *s*_*i*_ = 1..*S*.

**Figure 1:**
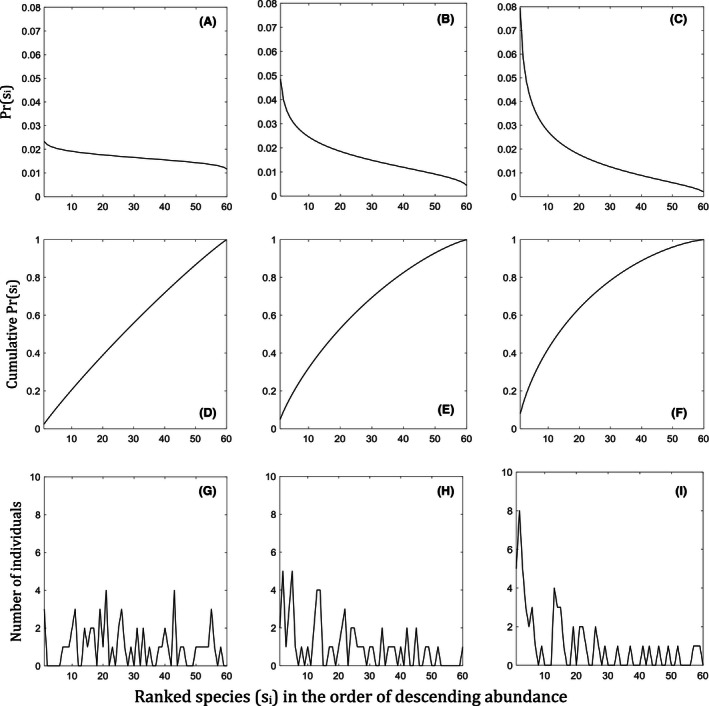
Three examples of simulated theoretical distributions of species assemblages using Equation (3) (A‐C) with coefficients of variation, CV, 0.15, 0.55, and 0.95, respectively. The number of species in the population, S, is 60, and the mean abundance is 1000 individuals. (D‐F): Cumulative distributions of equation (3) for the same assemblages. (G‐I): Examples of random samples of 100 individuals extracted from the respective assemblages.

As we assume that the likelihood of an individual of a species being caught is proportional to their abundance (a common assumption when species‐specific variation in the probability of selection is unknown), we can use Equation (3) to generate a random sample of individuals being caught from an assemblage for parameters *S* and σ. Figure 1 shows the theoretical outcomes of Pr(*s*_*i*_) of the model (Eq. (3)) and their cumulative distributions. In theory, the smaller the CV, the greater is the evenness of the ranked‐species abundance, and the larger the CV, the greater is the rate of decrease in the ranked‐species abundance (Fig. 1). A large CV results in large relative abundances of the most common species and a large number of rare species that are generally difficult to detect.

### 2.3 MCMC simulation method of sampling

We used the cumulative distribution in Equation (3) to sample individuals using MCMC from different species assemblages, characterized by parameters; species richness, *S*, mean abundance per species, and CV, by identifying species by their rank given by $\frac{s_{i}}{S}$ (between >0 and 1). Here, we calculated the cumulative density function of Equation (3) for species in the order of the most common, $\frac{1}{S}$, to the rarest, 1 (or, $\frac{S}{S}$), in the order *s*_*i*_ = 1…*S*. We generated random numbers between 0 and 1 from a uniform distribution (using the function *rand()* in MATLAB) which was matched with the value classes [(0:$\frac{1}{S}$), $\left(\frac{1}{S}:\frac{2}{S}\right)$,…,$\left(\frac{S-1}{S}:1\right)$) of the cumulative distribution and extracted the species (by its rank) corresponding to the matching class. With this approach, we can randomly draw any number of individuals from the assemblage with a distribution given by Equation (3) and identify which species are drawn by their rank. Note that to draw individuals from a known assemblage (statistical population) all that is required are the var*,* or CV and mean, of the SAD, and the number of species in the assemblage, *S*. Sampling occurred with replacement as recommended by Walther & Moore (2005), which also falls in line with the assumption in the derivation of the richness estimators (Gotelli & Colwell, 2011).

### 2.4 MCMC sampling from other SADs

We derived sampling algorithms for log series, geometric series and negative binomial SADs from their respective inverse cumulative density distribution functions following the same method that we proposed above for the lognormal distribution. We used MATLAB Statistical Tool Box. The negative binomial SAD, NB (*r*,*p*), is given by parameters *r* = mean/var and *p* = *rp*/(1−*p*). The log series SAD is given on the basis of the number of species written as $\alpha x^{n}/n$ with *n* number of individuals from 1…*n*, such that α and *x* (0 < *x *<* *1) are constants. The geometric series SAD is given on the basis of the number of species written as α*x* ^*n*^ with *n + *1 number of individuals for *n* = 0…*n*, such that *a* is the first term of the series, and *x* (0 < *x *<* *1) are constants.

### 2.5 Sampling Scenarios and species richness estimations

We used MATLAB (b) for programming mathematical and statistical models and to conduct all estimation procedures. For lognormal (LN) SAD, we simulated 6,000 species assemblages, each with abundance (*N*) of sampled population randomly varying between 10^3^ and 10^5^; species richness (*S*) between 1 and 500 (weighted by *N* × 10^−3^); sample size (*n*) between 300 and 5,000 individuals from the assemblages, such that *n* < *N*, for each fixed CV from 0.15, 0.25,.., to 1.15, using MCMC, satisfying the range of parameter values observed in ballast water (Appendix S1). We quantified the MSE of the abundance‐based estimators, resampling from each assemblage 1,000 times with replacement, for each CV, resulting in a total of 6.6 × 10^7^ samples. We used Chao1, Chao2, Jk1a, Jk2a, and Jk1i/2i formulae. We split each of the 1,000 single samples into two samples, randomly, 300 times, and estimated the mean richness given by the respective incidence‐based estimators. The mean of the estimates is slightly more accurate than the median of the estimates (Appendix S2). Thus, variance for the incidence‐based estimations in the MSE calculation is given by the summation of (1) the variance of the estimation over the 1,000 random draws, and (2) the variance of the estimates by 300 splits of each 1,000 single samples. The variance of the abundance‐based estimations in the MSE calculation is given by just the former. Appendix S1 gives the ranges of the above parameters as they apply to data from the ballast water samples. We repeated the same method with samples split into three and four samples also to test the same.

To examine both the trivial cases and the full range of the proportion of samples (1%–100%) from the population size, we repeated the same simulation experiments for 10,000 species assemblages from each of log series, geometric series, negative binomial and also lognormal SADs, with 500 repeated samples, each split randomly also into two samples 300 times, with *N* between 100 and 1,000; *S* between 2 and 20; *n* as a percentage of *N* from 1% to 100%, for each fixed CV from 0.15, 0.25,.., to 1.15.

## 3 Results

### 3.1 For lognormal SAD

**Figure 2:**
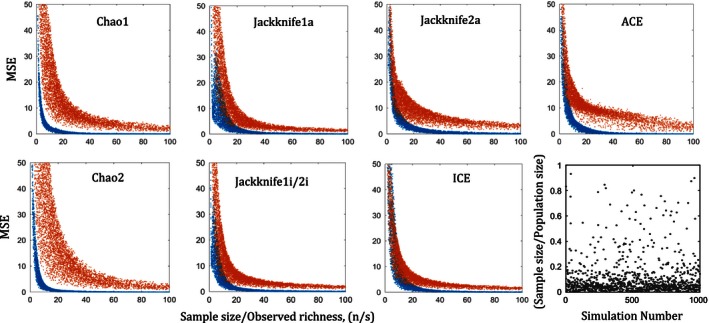
Mean‐squared error (MSE) of species richness estimators for lognormal SAD with respect to the ratio of sample size to observed richness (*n*/*s*), given coefficient of variation, CV, 0.35 (blue), and 0.95 (red). Simulations are based on 6,000 community assemblages having a lognormal species abundance distribution with population richness, *S*, selected randomly between 1 and 500 weighted by *N*; abundance (*N*) (103–105); and sample size (*n*) (300–5,000). Each MSE is estimated based on 1,000 repeated samples from each assemblage with replacement; Chao1, Jackknife1a, Jackknife2a, and ACE are based on a single abundance‐based sample, whereas Chao2, Jackknifek1i/2i, and ICE are based on a single sample randomly split into equally sized pairs of 300 samples using MCMC. Bottom right panel: simulated parameter space of sample size/population size

In general, the MSE decreases when the sample size to observed richness ratio, (*n*/*s*), increases, for all estimators; both abundance‐based with a single sample and incidence‐based randomly split into equally sized samples (Fig. 2). Figure 2 shows that the higher the coefficient of variation (CV) of the underlying log‐transformed species‐abundance distributions (SAD), the greater the MSE of the estimators. That is, the higher the number of rare species in a community assemblage, the greater the expected error of the estimators, on average. The decrease in MSE with increasing sample size to observed richness ratio, (*n*/*s*), converging to near zero, indicates that larger errors in estimation, as for uneven communities, can be offset by large samples. The rate of convergence of error (MSE) to zero is greater when CV is smaller. Note that in the bottom right corner panel of Fig. 1, the proportion of sample size to population size (in log‐scale) of the simulations is concentrated around 11% (the mean). This is most often the practical reality in field sampling. The cases where 1%–100% of the population is sampled in relatively small statistical populations of 100–1,000 individuals are presented later for theoretical interest.

**Figure 3:**
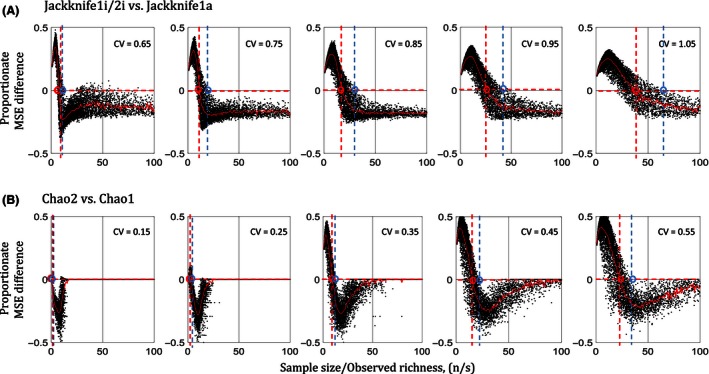
Proportionate difference in mean‐squared error (MSE) between (A) Jackknife1i/2i (with 2‐split samples) and Jackknife1a, that is, [Jackknife1i/2i‐Jackknife1a]/Jk, where Jk is min{Jackknife1i/2i, Jackknife1a}, for coefficient of variation CV > 0.65, and similarly (B) between Chao2 (with 2‐split samples) and Chao1, for CV < 0.65, with respect to sample size to observed richness ratio, (*n*/*s*). The circles on the intersections between dashed lines and *y*‐axis = 0 are the points beyond which the proportionate differences in MSE (on the *y*‐axis) are more likely (probability >0.5) to be negative (red circles), which we refer to as the mid‐criterion, and almost all negative (probability ~1) (blue circles), which we refer to as edge‐criterion. Beyond the points of red circles, where (*n*/*s*)_C_, on the *x*‐axis, the MSE of the Jackknife1i/2i and Chao2 is more likely to be less than Jackknife1a and Chao1, respectively. (Data are based on simulations as in Fig. 1)

**Figure 4:**
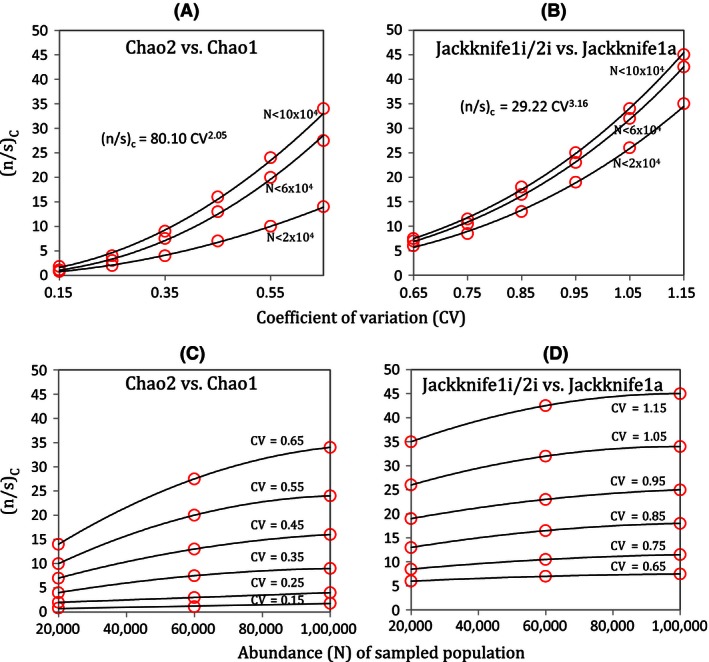
For a given combination of parameters, this figure illustrates whether the splitting method yields more accurate estimations, and if so, which estimator should be used (see Appendix S3 for specific examples). Shown are the critical sample sizes to observed richness ratio, (*n*/*s*)_C_, (red circles—the *mid‐criterion* from Fig. 3), modeled with respect to coefficients of variation (CV) for differences in MSE (A) between Chao2 (samples with two splits) and Chao1; and (B) between Jackknife1i/2i (samples with two splits) and Jackknife1a, for different ranges of abundances (*N*); (c10 & d) (*n*/*s*)_C_ modeled with respect to *N* of the sampled population calculated from graphs in panels (A & B), respectively. The best fit lines in panels (A) & (B) are power functions ((*n*/1 *s*)_C_ = 80.10 CV2.05, and (*n*/*s*)_C_ = 29.22 CV3.16 given for 103 < *N* < 105 for Chao2 and Jackknife1i/2i, respectively); and in panels (C) & (D) are polynomials to the degree 3. Although there are points of singularities on the CV‐axis in panels (A, B) for lower values of CV beyond the simulated ranges, we do not discuss them here as they do not impact our results given our choice of estimators. The modeled critical points (threshold equations) are valid also for other estimators with respect to Chao2 and Jackknife1i/2i (Fig. 5). Thus, given the graphs, for any given combination of *n*,* s*,* N*, and CV, one could determine should the splitting methods be used, and if so, which estimator be used; Chao2 or Jackknife1i/2i, depending on CV < 0.65 or >0.65, to obtain a higher propensity of a lower MSE

For CV > 0.65, the MSE of Jackknife1i/2i (for 2‐split samples) is lowest compared to that of all other estimators above a critical ratio of sample size (*n*) to observed species richness (*s*), (*n*/*s*), depending on the relative dispersion (CV) of the SAD, and the abundance (*N*) of the sampled statistical population. Out of all estimators, MSE estimates of Jackknife1a are the closest to the MSE estimates of Jackknife1i/2i. Thus, in Fig. 3, we show the proportionate differences between the MSE of Jackknife1i/2i and that of Jackknife1a, in order to measure the critical (*n*/*s*), denoted by (*n*/*s*)_C_. The (*n*/*s*)_C_ are the points above which the likelihood that “MSE of Jackknife1i/2i is lower than MSE of Jackknife1a” is the greatest, probability >0.5, which we call the *mid‐criterion* point (red circle), for respective CVs. Figure 4 shows the modeled (*n*/*s*)_C_ (from critical points extracted from Fig. 3) as a power function of CV, using the nonlinear least‐squared method, for different ranges of *N*. Thus, for any combination of (*n*/*s*) above the respective (*n*/*s*)_C_ for a given *N* and CV, the Jackknife1i/2i with 2‐split samples performs better, on average, than Jackknife1a with a single sample. The blue circles in Fig. 3 are the critical points (*n*/*s*) above which the likelihood that “MSE of Jackknife1i/2i is lower than MSE of Jackknife1a” is almost 100%. We call these *edge‐criterion* points, for respective CVs. For CV < 0.65, Figs 3 and 4 also show the case where Chao2 with 2‐split samples performs better than Chao1 with single sample, and the modeled (*n*/*s*)_C_ as a power function of CV, using nonlinear least‐squared method, for given ranges of *N*.

**Figure 5:**
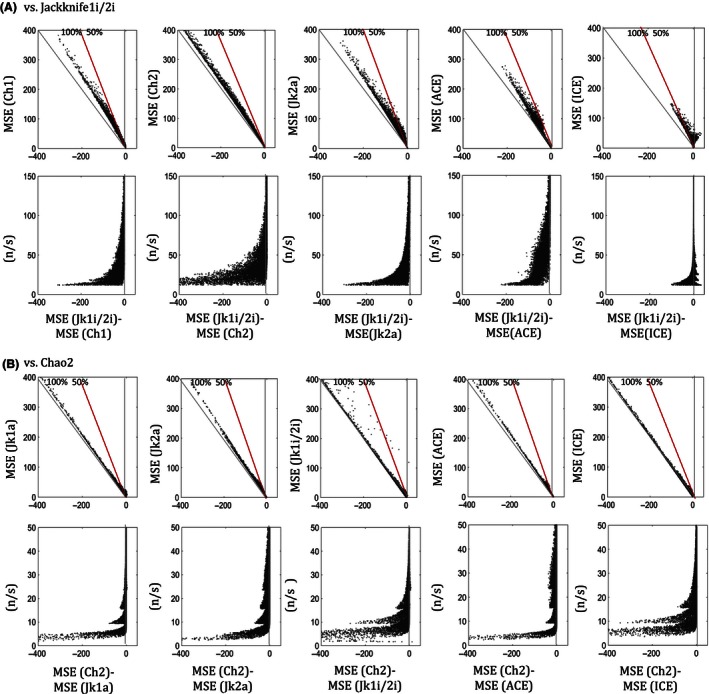
The mean‐squared error (MSE) of the estimators, and sample size to observed 1 richness ratio, (*n*/*s*), with respect to the difference in MSE between (A) Jackknife1i/2i and the other estimators, for CV > 0.65, and, (B) Chao2 and the other estimators, for CV < 0.65. Diagonal lines indicate the 100% (solid gray) and the 50% (solid red) reduction in MSE resulting from splitting method and using the given incidence‐based estimator; (A) Jackknife1i/2i, or (B) Chao2. (Data are based on simulations as in Fig. 1.). (ACE‐abundance‐based coverage estimators, ICE‐incidence‐based coverage estimators, Jk1a and Jk2a—1st‐ and 2nd‐order abundance‐based Jackknife, Jk1i/2i—1st‐ or 2nd‐order incidence‐based Jackknife, Ch1—Chao1, Ch2—Chao2)

The criterion (*n*/*s*)_C_, modeled with respect to Jackknife1i/2i vs. Jackknife1a, is also true for Jackknife1i/2i vs. all other estimators (Fig. 5). Figure 5A shows the absolute differences of the MSE between Jackknife1i/2i and all others estimators after filtering the samples based on the same (*n*/*s*) > (*n*/*s*)_C_ criterion for the given *N* and CV in Fig. 4. These figures suggest that Jackknife1i/2i performs better than all other estimators, in general, for Cv > 0.65. Based on Fig. 5B, Chao2 performs better than all other estimators, in general, for CV < 0.65.

Using Jackknife1i/2i and Chao2 estimators with randomly split samples of equal size, for CV > 0.65 and <0.65, respectively, can reduce the expected error (MSE) by up to 100% (Fig. 5). Figure 5 shows that improvement in the estimations by Jackknife1i/2i and Chao2 resulting from splitting is larger at lower (*n*/*s*), and also larger, the greater the MSE of the other estimators. In cases where (*n*/*s*) < (*n*/*s*)_C_, that is, (*n*/*s*) is lower than the critical values, Jackknife1a and Chao1 with single sample perform better for CV > 0.65 and CV < 0.65, respectively. When (*n*/*s*) increases, the difference in MSE between estimators converges to near zero for all estimators, while the MSE of individual estimators also tends to near zero (Fig. 5).

**Figure 6:**
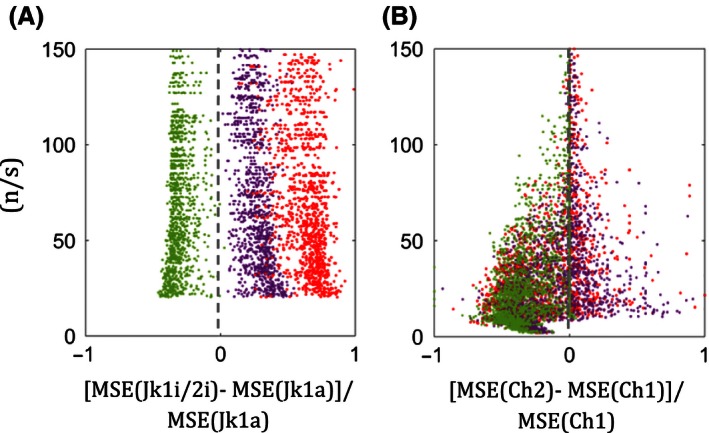
The sample size to species richness ratio, (*n*/*s*), with respect to proportionate differences in mean‐squared error (MSE) of (A) Jackknife1i/2i (with split samples) and Jackknife1a, and (B) Chao2 (with split samples) and Chao1 (Green dots). Purple and red dots indicate those with splitting samples into 3 and 4 random samples, respectively. (Data are based on simulation as in Fig. 2)

Furthermore, Fig. 6 shows the proportionate differences in MSE between Jackknife1i/2i and Jackknife1a, for 2, 3, and 4 random splits of samples for CV > 0.65 and 10^3^ < *N* < 10^5^. Jackknife1i/2i and Chao2 perform better than Jackknife1a and Chao1, for CV > 0.65 and CV < 0.65, respectively, but only when two random splits are carried out. Thus, splitting into two random, equally sized samples is the most reliable method for this proposed optimization. Examples for the application of the splitting method are given in Appendix S3.

**Figure 7:**
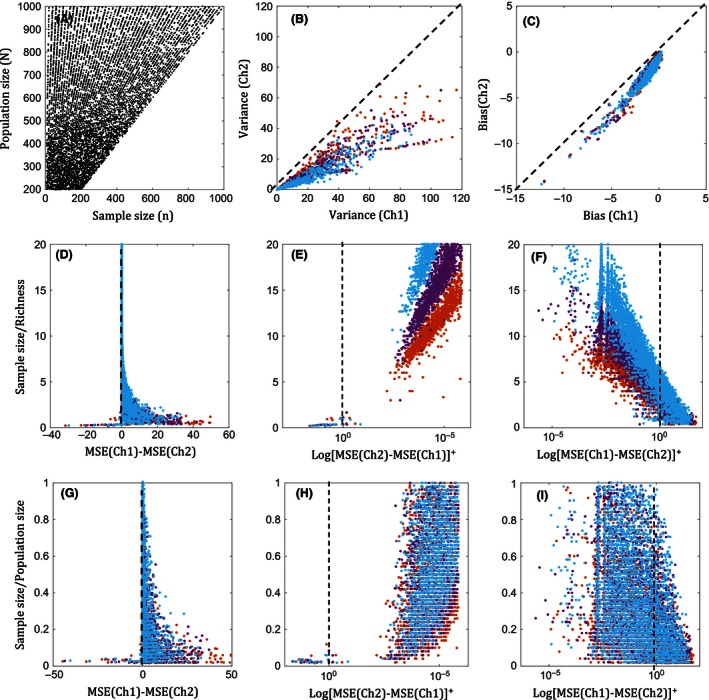
Comparison of mean‐squared error, MSE, of estimators based on 10,000 simulated assemblages with 500 repeated samples, covering 1%–100% of the populations *N*~100–1,000. Panel (A) is showing the simulated sample space. Richness (*s*) is between 2 and 20. [Brown, blue, purple] = [0.25, 0.45, 0.65 CV]. Panels (B‐C) are the comparison of bias and variances between Ch1 and Ch2 estimators. Panels (D‐F) and Panels (G‐I) are the sample size to richness ratio, and the sample size to population size ratio, respectively, with respect to the difference in MSE between Ch1 and Ch2. The above results are qualitatively similar for the cases between Jk1a vs. Jk1i/2i, and ACE vs. ICE. (Ch1—Chao1, Ch2—Chao2, Jk1a—1st‐order abundance‐based Jackknife, Jk1i/2i—1st‐ or 2nd‐order incidence‐based Jackknife)

Note that in Fig. 7, when the proportion of population sampled is relatively large, MSE of Chao1 has the propensity to yield values lower than MSE of Chao2 with split‐in‐two samples (d), although they are negligibly small (~10^−5^), and the MSEs of the estimators are converging to zero. Transformation of the *x*‐axis to log‐scale (E and F; H and I) highlights these subtle differences in the expected errors. This trivial phenomenon occurs when the sample size is large enough such that all the species are represented in the sample at least with doubletons, and thus, Chao1 yields the expected value, which is the population richness, whereas Chao2, with the sample split into two, is slightly positively biased. However, as we used sampling with replacement, there is always a possibility that Chao2 also outperforms Chao1 even at these sample sizes. This case was similar between other abundance‐based and incidence‐based estimators. In contrast, in cumulative sampling, where sampling is carried out until all the species are detected, the overestimations by Chao2, although negligibly small, consistently occur above a certain larger proportion of the population sampled (Appendix S4). Furthermore, panels (b) and (c) (Fig. 7) indicate that the biggest contributor to the difference in the MSE between Chao1 and Chao2 is the expected variance of the repeated samples, but not the expected bias. This mechanism is the same, also between the other given abundance‐based and incidence‐based estimators.

### 3.2 For other SADs

**Figure 8:**
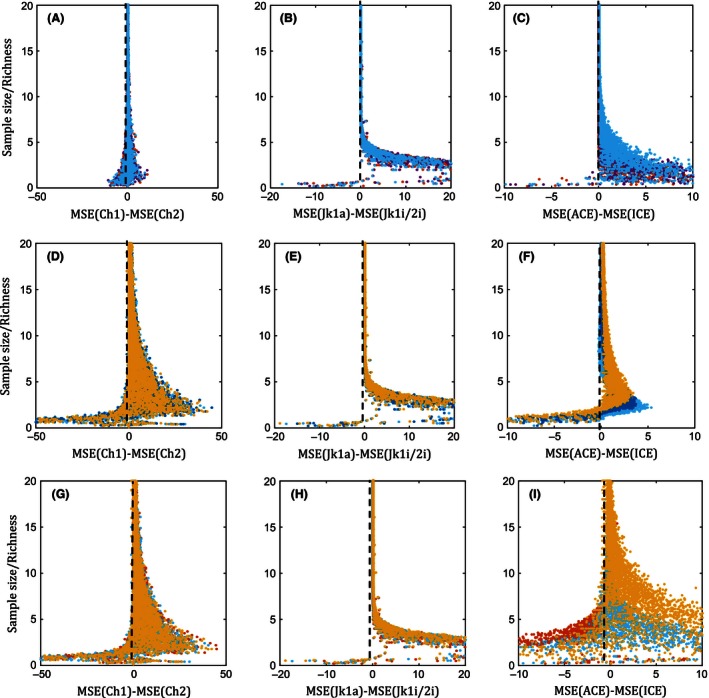
Comparison of mean‐squared error, MSE, between Chao1 vs. Chao2, Jk1a vs Jk1i/2i, and ACE vs. ICE, for 500 repeated samples from 1,000 population assemblages generated via geometric series (A–C), log series (D–F), and negative binomial (G–I) distributions with *N* = 1,000–10,000, *s* = 2–10, and *n* = 1%–100% of *N*. [Red, purple, light blue, dark blue, yellow dots] = [0.25, 0.45, 0.65, 0.85, 1.05 CV]. Ch1—Chao1, Ch2—Chao2, (Jk1—1st‐order abundance‐based Jackknife, Jk1i/2i—1st or 2nd‐order incidence‐based Jackknife)

Figure 8 shows the outcome of the splitting method when the sampled statistical populations are log series, geometric series, and negative binomial. Here, also we simulated samples 1%–100% of the population size. The figure indicates that the splitting method works well regardless of the qualitative differences in the forms of the ranked‐species‐abundance distributions, which are, in general, linear, and nonlinear declining functions of species ordered from the commonest to the rarest, with varying rates of decline.

## 4 Discussion

The accuracy of a richness estimation method indicates how likely an estimation is close to the population richness at random. We demonstrated that randomly splitting a species‐abundance sample into two equally sized samples and using an appropriate incidence‐based estimator increase the accuracy of the richness estimation substantially under certain criteria and conditions. Two splits may be optimal because the estimators that we used incorporate only the second‐ or lower‐order functions. The Chao2 estimator, with split samples, performs better when the relative dispersion parameter, the coefficient of variation, CV < 0.65, that is, when the community displays high evenness with few rare species, whereas Jackknife1i/2i, with split samples, performs better when CV > 0.65, that is, when the community exhibits a greater potential for rare species, more likely results in doubletons and singletons in an enumerated sample. For the splitting method to be more effective, the ratio of sample size to observed richness should be relatively small, but greater than a critical value, which depends on the estimated CV and the abundance (*N*) of the statistical population. Furthermore, the advantage of the splitting method is greater when the CV is large (>0.65); that is, when the likelihood of rare species in a population is large, in which case, Jackknife1i/2i is recommended. The Chao2 with split samples is recommended for smaller CV (<0.65). However, all richness estimators perform relatively well, in general, when a community has few rare species, as the sample contains greater coverage (Brose et al., 2003; Walther & Moore 2005). The splitting method yielded qualitatively similar results, also when species‐abundance distributions (SADs) are log series, geometric series, and negative binomial, as the ranked‐species‐abundance distributions of SADs, in general, are linear and nonlinear declining functions of species ordered from the commonest to the rarest. Thus, it appears that the splitting method is suitable for a large number of common SADs in ecology and is a logical alternative to increasing the sample size, commonly carried out in order to increase the accuracy of richness estimation.

When the sample size is large compared to the observed richness, the expected difference in errors between the estimators converges to near zero; that is, they become more similar, while expected errors of the individual estimators also converge to near zero. Although there can be an exchange of the better performing estimator at large sample sizes, the differences in expected errors between the estimators in such scenarios are negligibly small. Also because, obtaining field samples, close to sizes that allow the above trivial scenario to occur, is usually less probable due to logistical constraints, we suggest that, in general, the splitting method can be regarded as the most advantageous, under the given criteria and conditions. Our study supports the notion that different richness estimators will be appropriate depending on the underlying SAD and the practical constraints involved in sampling (Colwell & Coddington, 1995), but most of the most difficult scenarios (relatively small samples, large communities, or high richness) can benefit from our sample splitting method.

The practical value of the splitting method is that the coefficient of variation (CV) and abundance *N* can be easily estimated by the data while the observed species richness, *s*, and the sample size, *n*, are readily available. An example of how to use these summary statistics with the splitting method is given in Appendix S3, which involves zooplankton community data collected from samples of ballast water from ships. In Appendix S3, we demonstrate that the ranges of abundance *N*, CV, *s*, and *n*, for these zooplankton samples are well within the range of our simulations and may be wide enough to generalize our method for estimating richness in natural habitats as well.

Differences in the performance of species richness estimators under certain scenarios (e.g., low through high sample coverage, sample size to richness ratio, and CV of the community) have implications for designing abundance‐ vs. incidence‐based community sampling programs and the different kinds of sample processing involved. For example, when sample coverage is relatively high, the choice of incidence‐ vs. abundance‐based sampling can be based largely on the preference of the investigator, such as the ability to address secondary objectives with the data, as neither approach has drastic differences in bias reduction. However, when sample coverage is low (e.g., a limited number or organisms collected from large, species‐rich communities), incidence‐based sampling using the splitting method is strongly preferred. Incidence‐based sampling requires a single large sample that is randomly partitioned into a species discovery matrix across the two samples. There is a practical advantage associated with collecting incidence data, which saves the investigator from enumerating each individual once a species has been detected in a sample. Therefore, in addition to improving accuracy, the methods outlined here are likely to reduce sample processing time when potentially thousands of enumerations would have occurred to satisfy abundance‐based sampling.

In conclusion, selecting the most appropriate species richness estimator depends on the structure of the underlying SAD, the sample size, the observed population abundance, and the observed richness. When the sample size to observed richness ratio is low and CV is high, there is a much greater likelihood that a richness estimate will deviate from the true richness of communities. We provide a simple method to retain the greatest degree of estimation accuracy under such scenario when sampling resources are at a premium.

## Funding Information

Financial support was provided by an NSERC Discovery Grant to SAB, and NSERC Visiting Fellowships at Fisheries and Oceans Canada to HR and FC, supported by Transport Canada. D.A.R.D. was supported by funding from the Great Lakes Fishery Commission.

## Conflict of Interest

None declared.
