# Supplementary material for: Optimizing performance of nonparametric species richness estimators under constrained sampling
Source: Ecol Evol. 2016 Sep 22;6(20):7311–22. doi: 10.1002/ece3.2463 (PMC5513256; doi:10.1002/ece3.2463)
Supplement: Supplementary file 1 [file ECE3-6-7311-s001.docx]

**Supporting Information:**

**Appendix S1**: **In this section, we illustrate the parameter ranges and properties of the zooplankton community samples obtained from ballast water.**

To determine the type of probability model, that ballast water zooplankton communities follow, we tested 156 plankton samples taken from ships from West and East coasts of Canada and the Great Lakes, which include trans-oceanic and coastal voyages and those having exchanged or non-exchanged ballast water in the mid oceans (see Table. S1.1). Using Shapiro-Wilk’s test, recommended for sample size <50 (Legendre and Legendre, 2012), we tested the hypothesis that species abundance is not distributed lognormally for each sample. There was little evidence that SAD were not lognormal: p>0.05 in 96%, and, p>0.01 in 98% of the 156 samples (Fig. S1.1(a)). The range of coefficient of variation (CV) of the log-transformed data are given in Fig.S1.1(b), and the range of abundance of sampled populations are given in Fig.S1.1(c).

Frequency

(a)

(b)

P-value

Frequency

Coefficient of variation (CV)

Density (m^-3^)

(c)

Frequency

**Fig. S1.1.** (a) Evidence for lognormal species abundance distributions (SAD): in 96% of sampled populations, p>0.05; in 98% of sampled population p>0.01 based on Shapiro-Wilk’s test for 156 ballast water samples. (b) The frequency distribution of coefficient of variation (CV) of the same data. (c) Frequency of samples with respect to the estimates of abundance of individuals of the population sampled.

**Table S1.1.** Description of ballast water datasets used, including sampling region (Atlantic, Pacific or Great Lakes), voyage route (trans-oceanic, coastal or domestic), ballast water exchanges status (yes or no), and data source.

| **Sampling region** | **Voyage type** | **Ballast water exchange** | **Number of samples** | **Data source** |
| --- | --- | --- | --- | --- |
| Atlantic | Trans-oceanic | yes | 4 | Simard et al. 2011 |
| Atlantic | Trans-oceanic | no | 4 | Simard et al. 2011 |
| Pacific | Trans-oceanic | yes | 2 | DiBacco 2007 |
| Pacific | Trans-oceanic | no | 2 | DiBacco 2007 |
| Pacific | Trans-oceanic | yes | 2 | DiBacco 2007 |
| Pacific | Trans-oceanic | no | 2 | DiBacco 2007 |
| Pacific | Trans-oceanic | yes | 19 | DiBacco 2008a |
| Pacific | Coastal | yes | 18 | DiBacco 2008a |
| Pacific | Coastal | no | 15 | DiBacco 2008a |
| Atlantic | Trans-oceanic | yes | 22 | DiBacco 2009 |
| Atlantic | Coastal | yes | 22 | DiBacco 2009 |
| Atlantic | Coastal | no | 20 | DiBacco 2009 |
| Great Lakes | Trans-oceanic | yes | 9 | DiBacco 2008b |
| Great Lakes | Coastal | yes | 4 | DiBacco 2008b |
| Great Lakes | Domestic | no | 4 | Briski et al. 2012 |

**Appendix S2: Comparison of MSE corresponding to the mean and median of estimates in best performing incidence-based estimators with random 2 split equally sized samples**

(a) Jackknife1i/2i

(b) Chao2


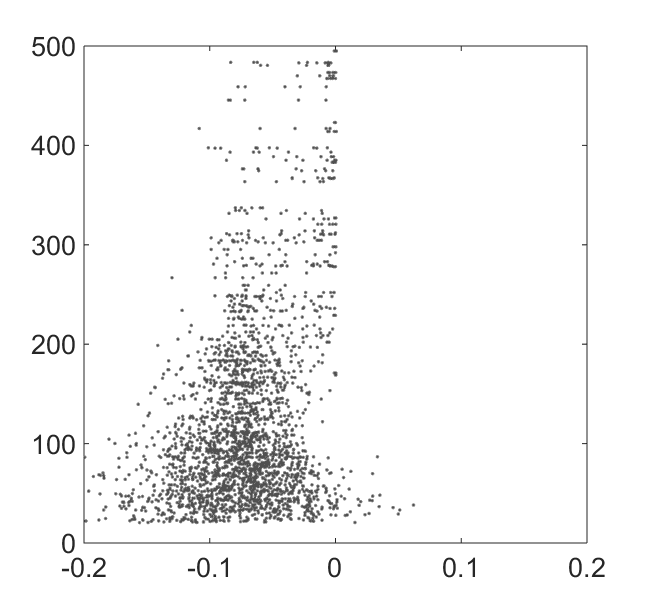

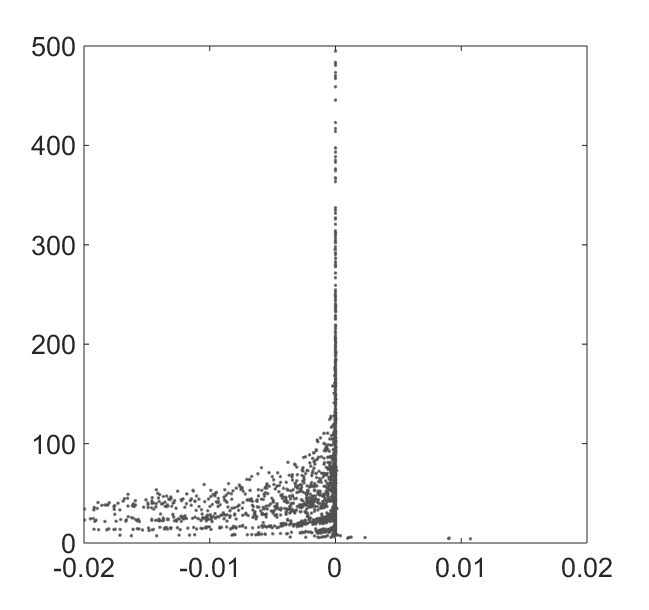


MSE (Mean)- MSE (Median)

(n/s)

**Fig. S2.1.** Difference in mean squared error (MSE) given by the mean and median of the estimates of (a) Jackknife1i/2i and (b) Chao2 estimations of the samples randomly split into two, 300 times. Data are from the same estimates as in Fig. 1, filtered by the edge-criteria.

**Appendix S3: Two examples of how to use the proposed method of splitting a sample in deciding the appropriate estimator for estimating species richness.**

Here we give two examples based on species abundance data (Appendix S1):

1. Data1(EC070 –Coastal exchanged): Density of species 1…12 observed species; (264, 32, 17, 3, 3, 2, 2, 1, 1, 1, 1, 1) in 33 liters.
2. Data2(EC058 –Coastal non-exchanged): Density of species 1…9 observed species; (357, 45, 17, 15, 5, 4, 2, 1, 1) in 6 liters.

From the given data, we can calculate the following parameters for the sampled statistical populations in 1m^3^:

| Data | s | n | CV | N | n/s |
| --- | --- | --- | --- | --- | --- |
| EC070 | 12 | 328 | 0.90 | 9840 | 27.3 |
| EC058 | 9 | 447 | 0.72 | 80460 | 49.7 |

where: s-observed number of species, n-total number of individuals belonging to all species in the sample (sample size), CV-coefficient of variation of log-transformed SAD, N-sampled population of which we estimate the true species richness.

*Decision Criteria:*

As CV>0.65 for both samples, the data-split method is appropriate using Jackknife1i/2i to estimate the population species richness, should the point (N, n/s) fall above the corresponding lines with respective to sample CVs in the graph below. If so, then Jackknife1i/2i will yield an estimate with greater accuracy than any other estimator. The case where the point is below the corresponding line for their CVs suggests that Jackknife1a is a better estimator, but not necessarily the best estimator among the all other estimators.

Thus, for Data1, we have (N, n/s)=(9840,27.3), (A); and for Data2, we have (80460,49.7), (B).

CV=0.65

CV=0.75

CV=0.85

CV=0.95

CV=1.05

CV=1.15

Observed abundance (N)

(n/s)_C_

B(80460,49.7)

A(9840,27.3)

The graph shows that for Data1, point A is far above the line corresponding to CV=0.90, and for Data2, point B is above the line corresponding to CV=0.72. Thus, this concludes that both data sets should be analysed using Jackknife1i/2i using the data-splitting method for higher accuracy.

*Estimation method:*

We split the sample randomly into two subsamples 1000 times. Thereafter we estimated the Jackknife1i/2i for each pair and calculated the mean of estimates. This also yielded a variance of the estimate, which we denote by Var1. Furthermore, we calculated the average variance associated with estimates of each of 1000 pairs, which we denote by Var2, as given by Manly (2006), in which we estimated the Jackknife1i/2i with one observation missing out each time, thus, for N-1 times. The overall confidence intervals (CI) are given by ±t_α=0.05,N-1_ ((Var1+Var2)/n)^0.5^.

The results are;

| Data | (Observed)  s | (Estimated)  Jk1i/2i±Var1±Var2 | 95% CI |
| --- | --- | --- | --- |
| EC070 | 12 | 15.3±0.5±0.1 | [15.3, 15.4] |
| EC058 | 9 | 10.4±0.3±0.1 | [10.4,10.5] |

The propensity for these estimates to be true in their respective populations is more than that provided by any other estimator, based on the theory developed in this paper.

**Appendix S4:**

1. **Theoretical examples of how splitting method (with Chao2) outperforms the traditional method (Chao1) for cases including the extreme cases –the large sample sizes (i.e., the sample Scenario 1 as below)**

Chao 2 with split samples Vs. Chao 1 with the single sample:

Here, we assume that a population has 5 species (spp.).

Chao1 is given by: $\text{S\_obs}+\frac{\left(n-1\right)}{\left(n\right)}\frac{\text{f}_{1}\left(f_{1}-1\right)}{2\left(\text{f}_{2}+1\right)}$

Chao2 is given by: $\text{S\_obs}+\frac{\left(m-1\right)}{\left(m\right)}\frac{\text{q}_{1}\left(q_{1}-1\right)}{2\left(\text{q}_{2}+1\right)}$

[by Chui et al. (2014).]. Here, S_obs –observed richness; n-sample size; f1-singletons; f2-doeubletons; q1-unique spp.; q2-common in both samples (in 2-split method); m=2;

We also assume that the first two species are relatively dense enough in the population that a sample of given size always captures more than doubletons (denoted by: >d) of the two species. And other species could be either doubletons (denoted by: d) or singletons (denoted by: s). And, furthermore the sample size is large enough that (n-1)/n~1.

Thus, take the following possibilities or the sample scenarios:

| Scenarios: | 1 | 2 | 3 | 4 | 5 | 6 | 7 |
| --- | --- | --- | --- | --- | --- | --- | --- |
| Spp1 | >d | >d | >d | >d | >d | >d | >d |
| Spp2 | >d | >d | >d | >d | >d | >d | >d |
| Spp | d | d | d | s | d | d | s |
| Spp | d | d | s | s | d | s | s |
| Spp | d | s | s | s | nd | nd | nd |

**nd -non-detected (but present in the population)

Now, we can draw the probability tree of the 7 above scenarios in the case of split samples (2 random sub-samples of equal size).

Note that:

1. ‘>d’ will always be found in both split samples thus results in contributing 1 value to q2;
2. ‘d’ will be either found in: either of the split samples with probability of 0.5 such that contributing 1 value to q2 (s.t. q1=0); or in one split sample, contributing 1 value to q1 (s.t. q2-0);
3. ‘s’ will be found in only one split sample, contributing to 1 value to q1 (s.t. q2=0);

For, e.g., the sample Scenario 2: [>d >d d d s]

|  | f1 | f2 | q1 | Prb. | q2 | Prb. |
| --- | --- | --- | --- | --- | --- | --- |
| Spp1 | 0 | 0 | 0 | 1 | 1 | 1 |
| Spp2 | 0 | 0 | 0 | 1 | 1 | 1 |
| Spp | 0 | 1 | 1 | 0.5 | 0 | 0.5 |
|  |  |  | 0 | 0.5 | 1 | 0.5 |
| Spp | 0 | 1 | 1 | 0.5 | 0 | 0.5 |
|  |  |  | 0 | 0.5 | 1 | 0.5 |
| Spp | 1 | 0 | 1 | 1 | 0 | 1 |

Thus, we can calculate the ‘expected value’ of Chao2, given the above table.

Summary calculations of the all given scenarios:

| Sample Scenario: | 1 | 2 | 3 | 4 | 5 | 6 | 7 | Bias* | Var* | MSE* |
| --- | --- | --- | --- | --- | --- | --- | --- | --- | --- | --- |
| Spp1 | >d | >d | >d | >d | >d | >d | >d |  |  |  |
| Spp2 | >d | >d | >d | >d | >d | >d | >d |  |  |  |
| Spp | d | d | d | s | d | d | s |  |  |  |
| Spp | d | d | s | s | d | s | s |  |  |  |
| Spp | d | s | s | s | nd | nd | nd |  |  |  |
| Chao2 | 5.1 | 5.2 | 5.3 | 5.5 | 4.2 | 4.3 | 4.5 | 0.13 | 0.28 | 0.29 |
| Chao1 | 5.0 | 5.0 | 5.5 | 8.0 | 4 | 4 | 5 | -0.21 | 1.82 | 1.86 |
| Diff in \|Bias_Ch1\|-\|Biar_Ch2\| | -0.1 | -0.1 | 0.2 | 2.5 | 0.2 | 0.3 | -0.5 |  |  |  |

*Note that we did not incorporate the probability of obtaining the particular Scenarios into the account of Bias, Var, and MSE.

The average bias of the estimates of these specific samples: Chao2=0.13; Chao1=-0.21; 🡪Chao1>Chao2

The variance of the estimates of these specific samples: Chao2=0.28; Chao1=1.82; 🡪 Chao1>Chao2

Mean square error of these special cases: MSE (bias^2+var): Chao2=0.29; Chao1=1.86; 🡪Chao1>Chao2

🡪 The larger the MSE, the lesser is the accuracy of the estimator.

This is the mechanism by which Chao2 with split samples becomes a more accurate estimator than Chao1 with the single sample, when sampled randomly.

Note that that Scenario 1 is the only case highlighted by the Reviewer 1; which is a special case. As a whole when sampled a population randomly, our results that we have shown in the manuscript are a fact.

Note that variance also gives a larger weight on the MSE. This was the same in our full and complete analysis given in the manuscript.

1. **Tractable simulation examples on how splitting method (with Chao2) out-performs the traditional method (Chao1) : Sequential (cumulative) sampling showing the cases above the large sample size threshold**

Here, we assume a population of (arbitrary) size 38 (individuals), consists of 5 species, forming 4 different distribution shapes [Cases: 1, 2, 3, 4) of Ranked Abundance Species frequency distributions, characterized by different CV (coefficient of variations).

Modeled Populations:

Case 1: Case 2:


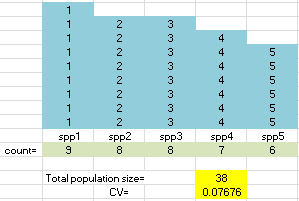

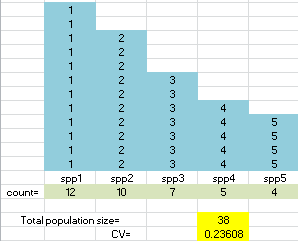


Case 3: Case 4:


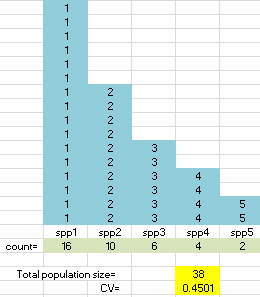

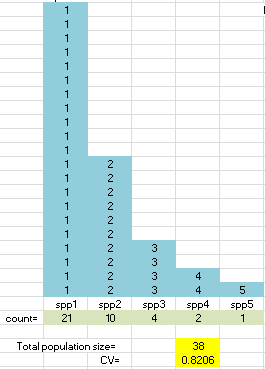


We assume that each individual is equally likely to be selected in a random sample regardless of the species type.

We generate random samples of 3 different sizes; 10, 20, 30; plus a random sample determined by the size which gives a ‘minimum of doubleton’ for each species (thus, 34 or 36, depending on the shape of SAD) (the case which Reviewer 1 highlighted).

For each sample we simulate 30,000 random samples.

We calculate Chao1 for each sample; and Chao2 for split samples.

The summary results:

|  |  | Sample size | Percentage (%) sampled from the population | Percentage of 30,000 estimates of which \|bias_Ch1\|>\|bias_Ch2\| | Percentage of 30,000 estimates of which \|bias_Ch1\|<\|bias_Ch2\| | Average  {\|Error Ch1\| -\|Error Ch2\|} |
| --- | --- | --- | --- | --- | --- | --- |
| Case 1 |  | 10 | 26 | 73.60 | 15.30 | 0.44 |
| CV=0.08 |  | 20 | 53 | 1.20 | 17.70 | -0.04 |
|  |  | 30 | 79 | 0.00 | 1.02 | 0 |
|  | *Threshold | 34 | 89 | 0 | 95.48 | -0.73 |
| Case 2 |  | 10 | 26 | 55.75 | 18.21 | 0.45 |
| CV=0.24 |  | 20 | 53 | 8.77 | 45.02 | -0.07 |
|  |  | 30 | 79 | 0.08 | 4.57 | -0.01 |
|  | *Threshold | 36 | 95 | 0 | 97.54 | -0.83 |
| Case 3 |  | 10 | 26 | 66.29 | 1.80 | 1.08 |
| CV=0.45 |  | 20 | 53 | 40.45 | 1.05 | 0.38 |
|  |  | 30 | 79 | 4.56 | 23.79 | -0.05 |
|  | *Threshold | 36 | 95 | 0 | 99.42 | -1.03 |
| Case 4 |  | 10 | 26 | 75.20 | 0.16 | 1.55 |
| CV=0.82 |  | 20 | 53 | 67.58 | 0.24 | 0.98 |
|  |  | 30 | 79 | 47.47 | 0.80 | 0.5 |

*The ‘threshold’ (critical) sample size above which all the spp. are doubletons or more; Total population size=38;

1. In all 4 cases (where the population distributions were of different shapes: CV =0.08; 0.24; 0.45; 0.82), Chao2 with split samples performed better with small sample sizes (consistently for 10; i.e., 26% of the population) w.r.t. both the overall bias (see the right most column of the above table, and the numbers highlighted in yellow). The MSE (mean squared error) follows the same.
2. At extreme critical sample size (>89% of the population size) (numbers in red), in which all species were minimum doubletons, Chao1 performed better w.r.t. Chao2 with split samples, considering the error alone.
3. This error of both Chao1 and Chao2 reduces with increasing sample size, converging to zero until the above extreme scenario is reached.
