# Supplementary material for: Optimizing performance of nonparametric species richness estimators under constrained sampling
Source: Ecol Evol. 2016 Sep 22;6(20):7311–22. doi: 10.1002/ece3.2463 (PMC5513256; doi:10.1002/ece3.2463)
Supplement: Supplementary file 3 [file ECE3-6-7311-s003.docx]

Pl. add to the supplementary:

**References:**

Briski, E., Wiley, C.J. & Bailey, S.A. (2012) Role of domestic shipping in the introduction or secondary spread of nonindigenous species: biological invasions within the Laurentian Great Lakes. *Journal of Applied Ecology*, **49**, 1124-1130.

Cordell, J.R., Lawrence, D.J., Ferm, N.C., Tear, L.M., Smith, S.S. & Herwig, R.P. (2009) Factors influencing densities of non-indigenous species in the ballast water of ships arriving at ports in Puget Sound, Washington, United States. *Aquatic Conservation: Marine and Freshwater Ecosystem*, **19**, 322-343.

DiBacco C. (2007) *Transpacific voyages 2007 data*. Canadian Aquatic Invasive Species Network. URL <http://www.isdm-gdsi.gc.ca/ais-eae/goHome-allerAccueil.do> [accessed 28 February 2013]

DiBacco C. (2008a) *West Coast 2007-2008 port data*. Canadian Aquatic Invasive Species Network. URL <http://www.isdm-gdsi.gc.ca/ais-eae/goHome-allerAccueil.do> [accessed 3 November 2014]

DiBacco C. (2008b) *Great Lakes port ballast water samples 2007-2008*. Canadian Aquatic Invasive Species Network. URL <http://www.isdm-gdsi.gc.ca/ais-eae/goHome-allerAccueil.do> [accessed 3 November 2014]

DiBacco C. (2009) *East coast port ballast water samples 2007-2009*. Canadian Aquatic Invasive Species Network. URL <http://www.isdm-gdsi.gc.ca/ais-eae/goHome-allerAccueil.do> [accessed 29 August 2014]

Manly, B.F. (2006) *Randomization, bootstrap and Monte Carlo methods in biology* (Vol. 70). CRC

Press.

Simard, N., Plourde, S., Gilbert, M. & Gollasch S. (2011) Net efficacy of open ocean ballast water exchange on plankton communities. *Journal of Plankton Research*, **33**, 1378-1395.

Sokal, R.R., & Rohlf, F.J. (1995) *Biometry* (3rd edn). pp. .WH Freman and Company. New York.
